# Transcriptional repressor NIR interacts with the p53-inhibiting ubiquitin ligase MDM2

**DOI:** 10.1093/nar/gkt1371

**Published:** 2014-01-10

**Authors:** Kristina Heyne, Juliane Förster, Roland Schüle, Klaus Roemer

**Affiliations:** ^1^José Carreras Research Center and Internal Medicine I, University of Saarland Medical Center, 66421 Homburg/Saar, Germany and ^2^Department of Urology, Center for Clinical Research, University of Freiburg, 79106 Freiburg, Germany

## Abstract

NIR (novel INHAT repressor) can bind to p53 at promoters and inhibit p53-mediated gene transactivation by blocking histone acetylation carried out by p300/CBP. Like NIR, the E3 ubiquitin ligase MDM2 can also bind and inhibit p53 at promoters. Here, we present data indicating that NIR, which shuttles between the nucleolus and nucleoplasm, not only binds to p53 but also directly to MDM2, in part via the central acidic and zinc finger domain of MDM2 that is also contacted by several other nucleolus-based MDM2/p53-regulating proteins. Like some of these, NIR was able to inhibit the ubiquitination of MDM2 and stabilize MDM2; however, unlike these nucleolus-based MDM2 regulators, NIR did not inhibit MDM2 to activate p53. Rather, NIR cooperated with MDM2 to repress p53-induced transactivation. This cooperative repression may at least in part involve p300/CBP. We show that NIR can block the acetylation of p53 and MDM2. Non-acetylated p53 has been documented previously to more readily associate with inhibitory MDM2. NIR may thus help to sustain the inhibitory p53:MDM2 complex, and we present evidence suggesting that all three proteins can indeed form a ternary complex. In sum, our findings suggest that NIR can support MDM2 to suppress p53 as a transcriptional activator.

## INTRODUCTION

NIR (novel INHAT repressor) is a predominantly nucleolar and partly nucleoplasmic ubiquitous repressor of transcription (1). INHATs (inhibitors of histone acetyltransferases) are multiprotein complexes of which the active moiety such as NIR blocks the acetylation of histones by the histone acetyltransferases p300/CBP and p300/CBP-associated factor (PCAF). This occurs probably through histone masking, i.e. association of the active subunits’ INHAT domain(s) with histone tails to preclude contact with p300/CBP or PCAF. INHAT subunits with histone masking ability are, for instance, the Set/TAF1β oncoprotein and pp32 (2,3), the polyglutamine-tract protein Ataxin-3 (4) and the corepressor PELP1 (5). Set/TAF1β and pp32 preferably bind to hypoacetylated histones. Acetylation of H3 and H4 inhibits INHAT binding (6,7); however, Set family INHATs are associated with histone deacetylases (HDACs) that can remove existing acetyl groups to restore the activity of the INHAT subunits and thereby support the suppressed state (6). NIR in contrast to the other known INHATs bears two INHAT domains (at the N- and C-terminus, respectively) and does not seem to coexist with HDACs (1). NIR is also unique among the known INHATs in that it is physically contacted and recruited to promoters by p53 (1) and p63 (8) where it acts as a potent inhibitor of gene transactivation.

p53 is a pleiotropic, homo-tetrameric transcription factor that is activated by mechanisms monitoring the functional integrity of a cell. Malfunction-induced activation often results in the physical elimination or silencing of a cell—e.g. by stimulation of apoptosis, senescence or differentiation—and is regarded as a central mechanism of tumour suppression (9,10). p53 is composed of N-terminal major and minor transactivation domains, a central DNA binding domain and C-terminal tetramerization and regulatory domain. Because p53 can act as a toxin, its function is tightly controlled.

The perhaps most important negative regulator of p53 is the multifunctional nucleoplasmic and partly cytoplasmic E3 ubiquitin ligase MDM2 (11–13). For example, MDM2 is crucial for restraining p53 during embryonic development (13); lack of MDM2 causes early p53-dependent apoptotic death of the mouse embryo (14,15). MDM2 acts at basically three levels: the ubiquitin-marking for degradation of p53, the export of p53 from the nucleus, and the direct transcriptional repression of p53-responsive promoters (16–20). The latter is attained through the inhibition by MDM2 of coactivator recruitment and through association of MDM2 with the 34 kDa subunit of TFIIE of the basal transcription machinery.

The interaction of p53 and MDM2 is posttranslationally regulated. Broadly, damage-induced phosphorylation of human p53 at threonine-18 weakens binding of MDM2, and phosphorylation at serines 15 and 20 facilitates the recruitment not only of transcriptional coactivators but also of the histone acetyltransferase p300/CBP (10,21,22). The latter binds to the N-terminus of p53 and acetylates histones, thereby opening up chromatin, and in addition, acetylates human p53 at lysine residues in the centre and the C-terminus (including K164, 370, 372, 373, 381, 382, 386) to prevent (re-)association with MDM2 (23). Here we present data indicating that NIR, in addition to binding to p53 (1), physically and functionally interacts with MDM2 and that it can support the MDM2-mediated repression of gene transactivation.

## MATERIALS AND METHODS

### Plasmids, chemicals and antibodies

pcDNA3-HA-Ubiquitin was purchased from Addgene. pcDNA-3.1 (+)-HA-MDM2, GST-MDM2 full length and GST-MDM2 deletion mutants were generated by polymerase chain reaction (PCR) and cloned into pGEX-4T1 (Amersham). Cloning details are available on request. MDM2 mutant D68A was kindly provided by Matthias Dobbelstein (Department of Molecular Oncology, Georg-August-Universität Göttingen, Germany) and pcDNA3.1 (+)-Flag-L11 by Karen Vousden (The Beatson Institute for Cancer Research, Bearsden, Glasgow, UK). Expression plasmids pCMX-Flag-NIR full length, pCMX-myc-NIR full length and NIR deletion mutants pCMX-myc-NIR (3–245), (147–609), (609–749) were constructed as reported previously (1). Aprotinin (# A1153), actinomycin D (# A1410), MG132 (# M7449), PMSF (# P7626), protease inhibitor cocktail (# P8345), 4′,6-Diamidin-2′-phenylindoldihydrochlorid (DAPI, # D9542), the HDAC-inhibitors trichostatin A (# T8552), sodium butyrate (# B5887) and nicotinamide (# N0636) were from Sigma, as were the ß-actin monoclonal antibody (clone AC-15, # A5441), the monoclonal anti-FLAG M2 antibody (# F3165), the monoclonal anti-glutathione-s-transferase antibody (clone GST-2, # G1160), the monoclonal anti-FLAG M2-Cy3 conjugate (clone M2, # F9594), the TRITC-conjugated anti-mouse IgG (# T5393), the monoclonal anti-HA-FITC (clone HA-7, # H7411), the monoclonal anti-HA-TRITC (clone HA-7, # H9037) and the peroxidase-conjugated secondary anti-mouse (# A9044) and anti-rabbit antibodies (# A6154), as well as all oligonucleotides. Anti-retinoblastoma (RB) monoclonal antibody was obtained from BD Pharmingen (# 554136) and anti-tubulin monoclonal antibody from abcam (ab7291). The rabbit anti-acetyl-lysine-antiserum (# 06-933) was purchased from Upstate; the rabbit monoclonal anti-acetyl-p53 (Lys382) antibody (# 04-1146) and anti-Mdm2-antibody 3G9 (# 04-1530) was from Millipore. The irrelevant monoclonal anti-HRS3-antibody as well as the polyclonal anti-transforming growth factor beta (TGF-β) receptor-antibody were kindly provided by Michael Pfreundschuh (Internal Medicine I, University of Saarland Medical Centre, Homburg, Germany), as was the anti-myc-antibody 9E10. Transfection reagent Nanofectin I (# Q051-005) was from PAA (Pasching, Austria), RNAifect (# 301605) from Qiagen. The anti-Mdm2 antibody IF2 (Ab-1, # OP46) was purchased from Calbiochem, as was the anti-p53-antibody DO-1 (# OP43L). Monoclonal anti-HA-antibody (HA.11 clone 16B12, # MMS-101P) was from Covance. Horse raddish peroxidase (HRP)-conjugated monoclonal anti-p53 antibody DO-1 (# sc-126) was from Santa Cruz Biotechnology. The secondary Alexa Fluor 488-labelled anti-rabbit IgG (# A11008) was from Invitrogen/Molecular Probes. Polyclonal anti-NIR antibodies (clone 2719 and 2910) were generated by one of us (R.S.; Centre of Clinical Research, University of Freiburg, Germany).

### Cell culture and transfection

All cells were maintained at 37°C in a 7% CO_2_ atmosphere. H1299 and HeLa cells were grown in Dulbecco's modified Eagle's medium (DMEM; #E15-843, PAA) with 10% fetal calf serum (#A15-101, PAA). U2OS cells were maintained in DMEM/Ham’s F-12 (# E15-813, PAA) with 10% fetal calf serum. HCT116 cells were grown in McCoy’s 5a medium with 10% fetal calf serum. For transient transfection, cells were seeded to reach 60–70% confluency at transfection and were transfected with Nanofectin I (PAA) following the manufacturer’s recommendations.

### Immunofluorescence analyses

In four Chamber Polystyrene Vessel Tissue Culture Treated Glass Slides (#354114, BD Falcon), 0.3 × 10^5^ H1299, U2OS or HeLa cells were seeded; 24 h after seeding they were transfected with the indicated plasmids. Cells were fixed in paraformaldehyde [4% in phosphate buffered saline (PBS)] for 10 min, permeabilized on ice with 0.2% Triton-X/PBS for 2 min, blocked for 30 min with 1 µg/ml bovine serum albumin (Merck) at 37°C and were then incubated with the indicated antibodies for 1 h. After several washing steps with PBS and 0.03% Triton-X/PBS, the nuclei (DNA) were stained with 0.2 µg/ml DAPI (4′,6-diamidin-2′-phenylindoldihydrochlorid, Sigma) in methanol. The slides were mounted with PermaFluorTM Aqueous mounting medium (#TA-006-FM, Thermo Scientific). A Leica DM IRB/E fluorescence microscope equipped with an Axio Cam colour camera (Zeiss) was used for microscopy; data were analysed with the Axio Vision 3.0 software.

### Coimmunoprecipitation and sequential immunoprecipitation

H1299 cells were seeded to ∼60% confluency and were transfected with the indicated plasmids with Nanofectin I (PAA) following the manufacturer’s recommendations. After 24 h, the cells were treated with 10 µM MG132 (Sigma) for 2–6 h. When acetylated proteins were precipitated, cells were also treated with 10 mM sodium butyrate, 5 mM nicotinamide and 1 nM trichostatin A for 4 h. Four micrograms of the indicated antibodies were added to a 1:1:1 mixture of protein A sepharose 4 Fast Flow (#17-5280-01), protein G sepharose 4 Fast Flow (#17-0618-01) and γ-Bind-sepharose (#17-0885-01, all GE-Healthcare) in coimmunoprecipitation (co-IP) lysis buffer (0.5% NP-40, 150 mM NaCl, 20 mM Na_2_HPO_4_ × 12 H_2_O, 5 mM EDTA, 10% glycerol, freshly supplemented with 1 mM PMSF and protease inhibitors) and incubated for at least 4 h on a rotating wheel at 4°C. Unbound antibody was removed by washing two to three times with co-IP lysis buffer. Cells were harvested and washed in sterile phosphate buffer saline. One-tenth of the cells was lysed in sodium dodecyl sulphate (SDS)-sample buffer (100 mM Tris–HCl (pH 6.8), 100 mM dithiothreitol (DTT), 4% SDS and 20% glycerol) and served as an input control. The remaining cells were permeabilized for 30 min on ice in co-IP lysis buffer. The extracts were then centrifuged at 16 000*g* for 30 min at 4°C. The supernatant was incubated with the sepharose-antibody-mix for at least 4 h at 4°C on a rotating wheel. Precipitates were washed three times with co-IP lysis buffer before SDS-sample buffer was added to the sepharose beads. All probes were boiled for 10 min and subjected to SDS-polyacrylamide gel electrophoresis (PAGE) for western blot analysis. For the co-IP of endogenous proteins, U2OS cells were seeded to 90% confluency and incubated with 10 or 100 nM ActD for 7 h before harvesting. To co-IP the endogenous proteins from U2OS cell nuclei, the same co-IP procedure was used, but with isolated nuclei. For this purpose, cells were resuspended in 300 µl of ice-cold hypotonic buffer A (10 mM HEPES pH 7.9, 10 mM KCl, supplemented with freshly added 1 mM PMSF and protease inhibitors) and incubated on ice for 15 min. Fifteen microlitres of NP-40 solution (10% NP-40 in H_2_O) was then added to reach a final concentration of 0.5%, the samples were vortexed immediately and centrifuged for 5 min at 800*g*. The pellet was the nuclear fraction; it was washed once with 1 ml of ice-cold buffer A. The nuclei were then resuspended in co-IP lysis buffer and processed as described above. Sequential immunoprecipitation of Flag-NIR/MDM2/p53 complexes was carried out as follows. H1299 cells were seeded in 4 × 10 cm dishes to ∼60% confluency and were transfected with the indicated plasmids with jetPEI® (Peqlab, #13-101-10 N) following the manufacturer’s recommendations. α-Flag antibody M2 (#F3165, Sigma) was covalently bound to Affi-Prep Hz hydrazide (#156-0015, Bio-Rad) as specified by the supplier. For equilibration of the matrix and removal of unbound antibody, beads were washed two to three times with co-IP lysis buffer (0.5% NP-40, 150 mM NaCl, 20 mM Na_2_HPO_4 _× 12 H_2_O, 5 mM EDTA) plus 2% glycerol. After 24 h, the cells were treated with 10 µM MG132 (Sigma) for 5 h. Cells were harvested and washed in sterile phosphate buffer saline. One-tenth of the cells was lysed in SDS-sample buffer (100 mM Tris–HCl (pH 6.8), 100 mM DTT, 4% SDS and 20% glycerol) and served as an input control. The remaining cells were permeabilized for 30 min on ice in co-IP lysis buffer plus 2% glycerol, followed by a centrifugation at 16 000*g* for 30 min at 4°C. The supernatant was incubated with 100 µl of the α-Flag antibody M2 bound to Affi-Prep Hz hydrazide overnight at 4°C on a rotating wheel. Precipitates were washed six times with co-IP lysis buffer plus 2% glycerol. Flag-conjugated complexes were eluted with 100 µl of FLAG peptide (#F3290, Sigma) overnight at 4°C on a rotating wheel. Two micrograms of each Mdm2 antibody (monoclonal anti-Mdm2-antibody 3G9 [#04-1530, Millipore] and polyclonal anti-Mdm2-antibody H-221 [#sc-7918, Santa Cruz Biotechnology]) as well as 2 µg of each irrelevant antibody (monoclonal anti-HRS3-antibody plus the polyclonal anti-TGF-β receptor-antibody) was added to a 1:1 mixture of protein A sepharose 4 Fast Flow (#17-5280-01), protein G sepharose 4 Fast Flow (#17-0618-01), in co-IP lysis buffer plus 2% glycerol and incubated overnight on a rotating wheel at 4°C. Unbound antibody was removed by washing two to three times with co-IP lysis buffer. Affi-Prep Hz hydrazide beads that were incubated overnight with FLAG peptide were centrifuged and supernatant was incubated with the Mdm2-antibody beads or with the irrelevant antibody beads, respectively. Incubation was overnight on a rotating wheel at 4°C. Precipitates were washed three times with co-IP lysis buffer plus 2% glycerol before SDS-sample buffer was added to the sepharose beads. All probes were boiled for 10 min and subjected to SDS-PAGE for western blot analysis.

### Protein extraction and western blot analysis

Cells were lysed in SDS-lysis buffer heated to 100°C, containing 100 mM Tris–HCl (pH 6.8), 100 mM DTT, 4% SDS and 20% glycerol. Protein (15–30 µg) was subjected to 8–13% SDS-PAGE and transferred to a PVDF membrane (Immobilon-P; #IPVH00010, Millipore). Signals were detected on overnight incubation of the membranes with one of the indicated antibodies (α-GST 1:1000; α-NIR 2910 1:1000, α-ß-actin 1:10000, α-Mdm2 clone 3G9 1:2000, α-Mdm2 clone IF2 1:200, α-Flag 1:10000, α-HA 1:1000, α-p53 DO-1 1:2000; α-myc 9E10 2 µg/ml), followed by a further incubation with a peroxidase-conjugated secondary anti-mouse (1:2000) or anti-rabbit (1:2000) antibody, and were detected by the Thermo Scientific ECL western blotting substrate (#32106) as specified by the supplier.

### GST pull-down assay

For GST pull-down analyses, equal amounts of GST, GST-MDM2 and GST-MDM2 deletion mutants, respectively, were immobilized on Gluthation-Sepharose beads (# 17-0756-01, GE-Healthcare), washed five times with GST low salt buffer (50 mM Tris–HCl, 200 mM NaCl, 0.8 mM EDTA, 0.1% NP40, 1 mM PMSF, 10 µg/ml aprotinin) and incubated with equal amounts of NIR full-length protein or NIR deletion mutants (*in vitro* translated from pCMX-myc-NIR full length or pCMX-myc-NIR (3–245), (147–609), (609–749). The *in vitro* translation was performed with the TNT-T7 Coupled Reticulocyte Lysate System (# L4610), according to the manufacturer’s protocol (Promega), with 1 µg of plasmid and ^35^S radiolabelled cysteine and methionine (Tran^35^S-Label™, # 51006, MP Biomedicals). After overnight incubation at 4°C, all probes were washed five times with GST high salt buffer (50 mM Tris–HCl, 500 mM NaCl, 0.8 mM EDTA, 0.1% NP40). GST protein complexes were eluted from the sepharose by adding SDS-sample buffer [100 mM Tris–HCl (pH 6.8), 100 mM DTT, 4% SDS and 20% glycerol] and by boiling samples for 10 min. The proteins were separated by SDS-PAGE, immobilized on PVDF membrane (Immobilon P, #IPVH00010, Millipore) and visualized by autoradiography. GST-proteins were detected with the monoclonal anti-glutathione-s-transferase antibody (clone GST-2).

### *In vivo* ubiquitination and MDM2 half-life determination

H1299 or U2OS cells were seeded in 10 cm dishes the day before transfection to reach a confluency of 70–80%. Cells were transfected with the indicated plasmids. After further 24 h, cells were treated with 10 µM MG132 for 4–6 h. Cells were then washed in cold PBS; one-tenth of the cells was saved as input control. The rest was lysed in 400 µl of TBS-lysis buffer (1% SDS in TBS) per 10-cm dish at 95°C for 5 min. Lysates were squeezed repeatedly through a 23 Gauge needle and vortexed vigorously for 10 s. Eight hundred microlitres of TBS-Triton buffer (1.5% Triton X-100 in TBS) per 400 µl of lysate was added and mixed before incubation with 100 µl of a 1:1 mix of protein G and protein A sepharose 4 Fast Flow (GE Healthcare), for 1 h on a rotating wheel at 4°C (preclearing). Samples were centrifuged for 5 min at max speed and supernatant was incubated with 100 µl of a 1:1 mix of protein G and protein A sepharose 4 Fast Flow preconjugated with 4 µg of the indicated antibody, for at least 4 h at 4°C on a rotating wheel. Samples were washed three times in 1 ml of cold TBS mix (1 part TBS-lysis buffer plus 2 parts TBS-Triton buffer); beads were resuspended in 30 µl of 95°C SDS-sample buffer (100 mM Tris–HCl (pH 6.8), 100 mM DTT, 4% SDS and 20% glycerol) and were boiled for 10 min. The proteins were separated by SDS-PAGE, immobilized on PVDF membrane (Immobilon P, Millipore) and detected by the indicated antibodies. For MDM2 protein half-life determination (CHX chase), H1299 cells were transfected with the indicated plasmids for 24 h and were then exposed to CHX (cycloheximide; 20 µg/ml) for 0–60 min, after which total protein extracts were prepared. The proteins were detected by standard western blotting.

### Reverse transcriptase-PCR and reverse transcriptase quantitative-PCR

Cells were seeded in 10-cm dishes and after 24 h were transfected with the indicated plasmids. Another 24 h later, cells were lysed in solution D (236.4 g of guanidium thiocyanate in 293 ml of water, 17.6 ml of 0.75 M sodium citrate, pH 7.0, and 26.4 ml of 10% sarcosyl, 0.72% 2-mercaptoethanol). Lysate was harvested and 0.1 ml of 2 M sodium acetate, pH 4.0, 1 ml of water-saturated phenol (Roth) and 0.2 ml of chloroform-isoamylalcohol (49:1) were added, mixed and cooled on ice for 15 min. After centrifugation (10 000*g*, 20 min, 4°C), the aqueous phase was collected and precipitated with isopropanol at −20°C overnight. After a further centrifugation (10 000*g*, 20 min, 4°C), RNA was dissolved in solution D and precipitated with isopropanol at −20°C for 1 h. The pellet was washed in 70% ethanol and dissolved in DEPC-water. The RNA was digested with RNase-free DNase I (#10776785001, Roche) for 60 min at 37°C, and 4 µg was used for the first-strand cDNA synthesis with SuperScript™III (#18080-093, Invitrogen, USA) as specified by the manufacturer. Semi-quantitative reverse transcriptase-PCR (RT-PCR) analysis was performed with AmpliTaq^R^ Gold DNA polymerase (#N808-0242, Applied Biosystems), using the following primers: *NIR* (for: cagctggtgtcctgtctgtc; rev: gcagtgcacatactgccagt; T_A_: 64°C); *MDM2* (for: atcgaatccggatcttgatg; rev: tcttgtccttcttcactaaggc; T_A_: 58°C), *gapdh* (for: tggtatcgtggaaggactcatgac; rev: agtccagtgagcttcccgttcagc; T_A_: 64°C). Quantitative RT-PCR analysis for *MDM2, NIR, p21* and *gapdh* was performed with the LightCycler® FastStart DNA Master SYBR Green I from Roche (#12239264001, Mannheim, Germany) using the *MDM2, NIR* and *gapdh* primers described above, and the following primers for *p21*: (for: ggcggcagaccagcatgacagatt; rev: atgaagccggcccacccaacctc). *NIR* and *gapdh* primers—T_A_: 60°C, final concentration of primers: 0.5 µM, final MgCl_2_ concentration: 2 mM; *MDM2* and *p21* primers—T_A_: 62°C, final concentration of primers: 0.5 µM, final MgCl_2_ concentration: 2 mM.

### Short interfering RNA-mediated knockdown

Short interfering (si) RNAs specific for MDM2 (Hs_MDM2_5—sense: UCAUCGGACUCAGGUACAUTT; antisense: AUGUACCUGAGUCCGAUGATT); and NIR (NIR1—sense: r(GACCUGAACUUCCCAGAGA)dTdT; antisense: rUCUCUGGGAAGUUCAGGUC)dTdT; NIR2—sense: r(GACAGGAAGGAUGAAGACA)dTdT; antisense: r(UGUCUUCAUCCUUCCUGUC)dTdT), were used to silence gene expression. As a control, an irrelevant siRNA (control—sense: r(UUCUCCGAACGUGUCACGU)dTdT; antisense: r(ACGUGACACGUUCGGAGAA)dTdT) was used. All siRNAs were purchased from QIAGEN. Exponentially growing cells were transfected with siRNA (40 nM) by RNAifect as recommended by the supplier (Qiagen).

### Reporter gene analysis

In 24-well-dishes, 2 × 10^5^ H1299 or U2OS cells were seeded. After 24 h, cells were transfected with Nanofectin I with the plasmid combination described in the figure legends. After further 24 h, cells were harvested. Luciferase assays were carried out with the Luciferase Assay System (#1500, Promega) as specified by the manufacturer.

## RESULTS

We showed previously that NIR, an inhibitor of histone acetyltransferases (INHAT), can bind to and repress the transactivator functions of the transcription factors p53 and p63 by inhibiting histone acetylation at promoters (1,8). In the course of these studies we had noticed that most cellular NIR is sequestered in the nucleolus and hence unavailable for p53 repression but that NIR is released into the nucleoplasm in response to the nucleolar stress imposed on cells by the chemotherapeutic drug actinomycin D (ActD). This finding seemed to be at variance with NIR acting as a repressor of p53 since ActD was known to activate rather than repress p53 (24). Another finding seemingly at variance with NIR being a repressor of p53 was that overexpression of NIR can enhance the p53 protein level. A potential solution to these contradictions could include MDM2, the major negative regulator of p53 function and stability. We hypothesized that NIR, like several other nucleolar proteins including ribosomal protein L11 (25–27), might interact directly with this p53-inhibiting ubiquitin ligase.

### NIR can colocalize with and bind to MDM2

**Figure 1.**
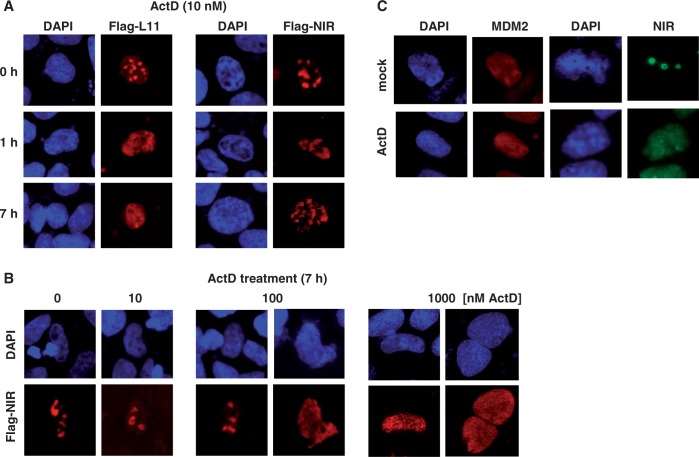
NIR translocates from the nucleolus to the nucleoplasm on ActD-induced nucleolar stress. (**A**) Translocation of Flag-L11 and Flag-NIR. H1299 cells were transfected with 0.5 µg of plasmids expressing Flag-L11 or Flag-NIR. At 24 h after transfection and treatment of the cultures with Actinomycin D (ActD) for the indicated times (h), cells were fixed, permeabilized and stained with Cy3-conjugated anti-Flag antibody (1:250; red stain) or nuclei were visualized by DAPI staining (blue). Punctual pattern indicated nucleoli; typically these were spared in DAPI stains. Diffuse pattern indicated nucleoplasm. (**B**) NIR requires elevated levels of ActD to leave the nucleoli. H1299 cells were transfected with 0.5 µg of Flag-NIR plasmid for 24 h, and Flag-NIR/nuclei were visualized as before. Here, cells had received increasing ActD doses for a duration of 7 h. Cells with nucleoplasmic Flag-NIR were present only at a dose of ≥100 nM ActD. (**C**) ActD-induced nucleolar stress caused endogenous NIR to translocate from the nucleoli to the nucleoplasm; endogenous MDM2 was nuleoplasmic regardless of ActD treatment. Exponentially growing U2OS cells (containing detectable levels of MDM2) were mock-treated or treated with 100 nM ActD for 7 h before fixation, permeabilization and incubation with polyclonal anti-NIR antibody 2910 (1:100) or monoclonal anti-MDM2 antibody 3G9 (1:100). Bound antibodies were visualized with secondary Alexa Fluor 488-conjugated anti-rabbit (1:7000) or TRITC-conjugated anti-mouse antibody (1:100). Nuclei were stained with DAPI.

We began our studies by investigating where in the cell ectopic L11 and NIR, both with a Flag-tag, localize and how these proteins respond to the nucleolar stressor ActD. When human H1299 lung adenocarcinoma cells (p53-deficient, low MDM2 levels) were transfected with plasmids producing Flag-L11 or Flag-NIR for 24 h, both proteins were primarily localized in the nucleoli, consistent with previous findings (8,25). On treatment with low-dose ActD (10 nM) for 0, 1 or 7 h, the characteristic nucleolar staining produced by Flag-L11 vanished and a diffuse nucleoplasmic staining became visible as early as 1 h after treatment (Figure 1A, left panels). In contrast, Flag-NIR’s predominantly nucleolar localization was unaffected by low-dose ActD (Figure 1A, right panels). However, higher doses of ActD (≥100 nM) caused Flag-NIR to leave the nucleolus (Figure 1B). Thus, although both Flag-L11 and Flag-NIR localize to the nucleoplasm in ActD-stressed cells as reported before (8,25), the respective triggering mechanism appear to be different and could be linked to one of the distinct effects known to be caused by different sublethal doses of ActD (24). Endogenous NIR detected by our polyclonal anti-NIR antibodies 2910 or 2719 (1,8) was also predominantly nucleolar in all tested cell types (H1299, U2OS, HeLa, 293T and various murine primary cells *in situ*). Figure 1C exemplifies the primarily nucleolar localization of endogenous NIR, as well as the nucleoplasmic localization of MDM2 in human U2OS osteosarcoma cells (p53-proficient, high levels of MDM2 relative to H1299 cells). As with ectopic Flag-tagged NIR, endogenous NIR translocated to the nucleoplasm in response to ActD treatment, whereas the MDM2 location remained unchanged.

**Figure 2.**
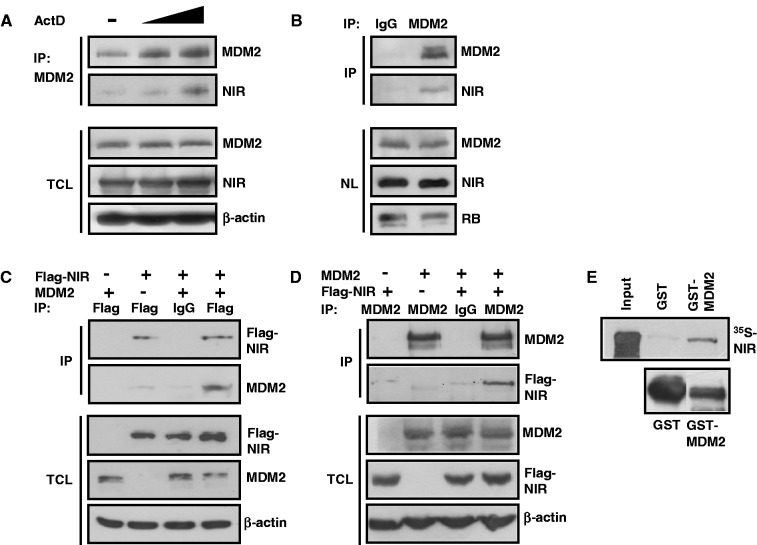
NIR binds MDM2 *in vitro* and *in vivo*. (**A**) Coimmunoprecipitation of NIR with MDM2 on ActD-induced colocalization of both proteins. U2OS cells were either mock-treated (−) or were incubated with 10 or 100 nM ActD for 7 h. MDM2 was immunoprecipitated from total cell extracts with monoclonal antibody 3G9. Standard western immunoblotting with MDM2 antibody 3G9 (1:2000), anti-NIR polyclonal antibody 2910 (1:1000) or anti-β-actin antibody (1:10 000) revealed the levels of each protein in the total cell lysate (TCL) and coimmunoprecipitation of NIR with MDM2. (**B**) Interaction of NIR and MDM2 in cell nuclei. As in (A), U2OS cells were treated with 100 nM ActD for 7 h. Cell nuclei were then prepared and MDM2 was immunoprecipitated from the nucleoplasm either with irrelevant IgG or MDM2-antibody 3G9. Proteins were detected as described in (A). Monoclonal anti-RB antibody for the detection of nuclear retinoblastoma protein as loading control was used at 1:1000. NL, nucleus lysate. (**C**) Coimmunoprecipitation of MDM2 with Flag-NIR. H1299 cultures were transfected with the indicated combinations of plasmids producing Flag-NIR (4 µg) and MDM2 (4 µg). At 24 h after transfection, cell extracts were incubated with anti-Flag antibody or irrelevant IgG. Immunoprecipitates, coimmunoprecipitates and the protein levels in the total cell lysate (TCL) were analysed by western blotting, as in (A). (**D**) Coimmunoprecipitation of Flag-NIR with MDM2. Transfections were performed as in (C). At 24 h after transfection, cell extracts were incubated with anti-MDM2 antibody 3G9 or irrelevant IgG. Immunoprecipitates, coimmunoprecipitates and the protein levels in the total cell lysate (TCL) were again analysed by western blotting. (**E**) NIR binds MDM2 in an *in vitro* GST pull-down assay. *In vitro* translated, ^35^S-labelled NIR is retained by bacterially expressed GST-MDM2 but not GST alone. The lower panel shows the expression of the GST proteins in bacteria. One-tenth of the labelled NIR was used as input control.

**Figure 3.**
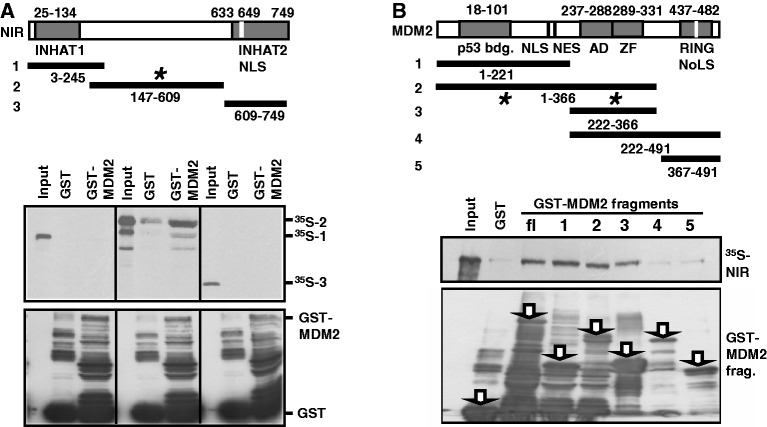
Mapping of the *in vitro* NIR:MDM2 interaction domains. (**A**) MDM2 is contacted by the central domain of NIR in GST pull-down assays. ^35^S-labelled fragments of NIR (1, 2, 3) were incubated with beads loaded with GST-MDM2 or GST alone. Only fragment 2 of NIR (asterisk) was retained by GST-MDM2. The lower panels show GST protein expression in bacteria. INHAT1, 2 depicts the respective domains; NLS, nuclear localization signal. Numbers indicate amino acid residues. (**B**) NIR is contacted by the N-terminal domain and the central acidic and zinc finger (AD/AZ) domains of MDM2 (asterisks) in GST pull-down assays. Pull-downs were performed as described in (D), by incubating ^35^S-labelled full-length NIR with the indicated GST-MDM2 fragments (fl = full length; 1, 2, 3, 4, 5). p53 bdg., major p53 binding domain; NLS, nuclear localisation signal; NES, nuclear export signal; RING domain; NoLS, nucleolar localization signal.

Next we asked whether NIR and MDM2 can physically interact with one another. In a first experiment, cultures of U2OS cells were mock-treated or treated with 10 or 100 nM ActD for 7 h. MDM2 was then immunoprecipitated from the cell extracts, and coimmunoprecipitation of NIR was analysed by standard western immunoblots. NIR coimmunoprecipitated with MDM2 in an ActD dose-dependent manner (Figure 2A). Similarly, NIR coprecipitated with MDM2 when MDM2 was precipitated from isolated H1299 cell nuclei (Figure 2B), suggesting that both proteins can be part of a common complex when colocalizing in the nucleoplasm. To corroborate this finding, p53-negative H1299 cells were transfected with combinations of plasmids producing Flag-NIR and MDM2. p53-negative cells were chosen because they typically harbour only little endogenous MDM2 and because NIR-MDM2 coprecipitation would be less likely indirect in the face of the fact that both NIR and MDM2 can associate with p53 through different domains (1,8,28,29). Immunoprecipitation at 24 h after transfection of Flag-NIR coprecipitated MDM2 (Figure 2C); *vice versa*, immunoprecipitation of MDM2 coprecipitated Flag-NIR (Figure 2D). To further study whether the interaction is direct, and to map the interaction domains, GST pull-down assays were performed. First, a GST pull-down with full-length human MDM2 fused to GST, and with *in vitro*-translated ^35^S-labelled full-length NIR, was carried out. GST-MDM2 _1__–__491_, but not GST alone, retained ^35^S-NIR _1__–__749_ (Figure 2E), suggesting that NIR and MDM2 bind directly to one another. GST pull-down analyses with radioactively labelled NIR fragments incubated with GST-MDM2 (Figure 3A), or with radioactively labelled full-length NIR incubated with GST-MDM2 fragments (Figure 3B), suggested that the central portion of NIR (residues 147–609) contacts the N-terminal domain (residues 1–122) and the central acidic and zinc finger domains (residues 222–366) of MDM2 *in vitro.* The two INHAT domains of NIR and the C-terminal RING domain of MDM2 seemed to be dispensable for the interaction. Combined, these results thus indicate that NIR and MDM2 can associate *in vitro* and *in vivo*.

### NIR can affect MDM2’s ubiquitination and stability

**Figure 4.**
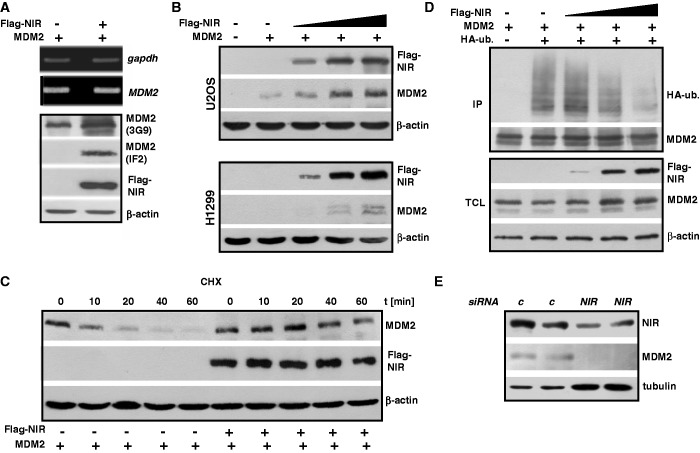
Ectopic NIR stabilizes MDM2 and inhibits MDM2 ubiquitination. (**A**) NIR causes MDM2 levels to increase independently of p53. H1299 cells were transfected with plasmids producing Flag-NIR (4 µg) and MDM2 (4 µg), and 24 h after transfection, total protein and RNA were extracted. The levels of *gapdh* and *MDM2* transcripts were determined by semi-quantitative RT-PCR (upper panels). Proteins were detected by western blotting and incubation with anti-Flag monoclonal antibody (1:10 000), monoclonal MDM2 antibodies 3G9 (1:2000) or IF2 (1:200), or β-actin antibody (1:10 000). (**B**) Flag-NIR increases MDM2 levels in U2OS and H1299 cells in a dose-dependent manner. U2OS and H1299 cultures were transfected with plasmids expressing Flag-NIR (0.5, 1.5 or 3.0 µg) and MDM2 (2 µg) as indicated. At 30 h after transfection, total protein was prepared and subjected to standard western blot analysis. Proteins were visualized as in (A), with antibody 3G9 for the detection of MDM2. (**C**) MDM2 has a longer half-life in the presence of Flag-NIR. H1299 cells were transfected with *MDM2* plasmid (0.5 µg) and *Flag-NIR* plasmid (1.5 µg) as shown. At 24 h after transfection, *de novo* protein synthesis was blocked by cycloheximide (CHX; 20 µg/ml) for the indicated times (CHX chase) and the levels of MDM2 and Flag-NIR were analysed by western blotting as in (A). (**D**) Flag-NIR inhibits the ubiquitination of MDM2 in a dose-dependent manner. H1299 cells were transfected for 24 h with the indicated combinations of expression plasmids to produce MDM2 (3 µg), Flag-NIR (0.5, 1.5 or 3.0 µg) and HA-ubiquitin (3 µg), and were then treated with the proteasome inhibitor MG132 (10 µM) for another 4 h. MDM2 was immunoprecipitated with antibody 3G9 from denatured cell extracts. The slower migrating bands indicative of HA-ubiquitinated MDM2 were detected by western blotting with monoclonal anti-HA antibody (1:1000); the remaining signals were detected as in (A). TCL, total cell lysate. (**E**) Knockdown of endogenous NIR reduces MDM2 protein level. H1299 cells were transfected with *control siRNA (c)* or a 1:1 mixture of *NIR siRNA1* and *2* (40 nM) for 42 h. Total protein extracts were analysed by western blotting with polyclonal anti-NIR antibody 2719 (1:1000), monoclonal MDM2 antibody 3G9 (1:2000) and monoclonal anti-tubulin antibody (1:1000).

We had previously noticed that ectopic NIR can enhance the level of MDM2 protein independently of p53. To further elucidate this, H1299 cells were transfected to express either MDM2 alone or a combination of MDM2 and Flag-NIR for 24 h. Although the levels of *MDM2* transcript were similar in these transfected cells, there was a robust increase in MDM2 protein in the cultures coexpressing MDM2 and NIR (Figure 4A), as assessed by western blotting with mouse monoclonal MDM2 antibody 3G9 whose epitope is not known to be sensitive to posttranslational modification (30). Mouse monoclonal MDM2 antibody IF2 failed to detect MDM2 in H1299 cells altogether, most likely because in contrast to 3G9, IF2 recognizes an epitope on MDM2 that is masked by posttranslational modification (30). Intriguingly, MDM2 was detected by IF2 when NIR was present (Figure 4A), suggesting that NIR can protect MDM2 from chemical modification(s) capable of masking the IF2 epitope. Flag-NIR was able to increase the MDM2 levels in U2OS and H1299 cells in a dose-dependent manner (Figure 4B). Combined, these findings suggested that ectopic NIR can stabilize MDM2.

MDM2 typically has a short half-life in cells because of ubiquitination and breakdown through the 26 S proteasome (31). We reasoned that if ectopic NIR can increase the level of ectopic MDM2 it might do that through the increase of MDM2’s half-life. Next we therefore performed cycloheximide (CHX) chase experiments by transfecting H1299 cells with combinations of plasmids producing Flag-NIR and MDM2 and assessing the protein levels after blocking the *de novo* protein biosynthesis for 0–60 min. As expected, MDM2 had a short half-life of ≤10 min in these cells. However, when Flag-NIR was coexpressed, the half-life of MDM2 increased to >40 min (Figure 4C). This increase could have been due to NIR affecting the ubiquitination of MDM2. To study this, we again cotransfected cells to express MDM2 alone or MDM2 plus Flag-NIR, and included in the transfections a plasmid producing HA-tagged ubiquitin. We then immunoprecipitated MDM2 from denatured cell extracts to preclude the detection of ubiquitin signals that stem from coprecipitating ubiquitinated MDM2 binding partners. The subsequent western blot analysis therefore detected only HA-ubiquitin that was covalently linked to MDM2. In accordance with the increased half-life of MDM2 in the presence of NIR, we found that NIR was able to inhibit the ubiquitination of MDM2 in a dose-dependent manner (Figure 4D). Conversely and consistent with these immunoprecipitations, when HA-ubiquitin was precipitated from the denatured cell extracts with anti-HA antibody and the precipitate was analysed with MDM2 antibody 3G9, only little MDM2 was detected in the presence of Flag-NIR (data not shown). Finally, we decided to test whether the reduction of endogenous NIR protein, through the incubation of H1299 cells with a mixture of two verified NIR-specific siRNAs, would exert any effect on the level on the endogenous MDM2 protein. Figure 4E shows the results of two experiments. When the cells were treated with the NIR siRNAs versus control siRNA for 42 h, the level of NIR decreased and concomitantly, the level of MDM2 decreased to below the detection limit. It should be noted though that this effect was detectable only in p53-deficient cells, whereas in cells with wild-type p53, knockdown of NIR did not reduce the MDM2 level. However, this finding is entirely consistent with NIR being a repressor of p53 (1) and with p53 acting as a transactivator of the *MDM2* gene (13). In sum, these results suggest that NIR can stabilize MDM2 through the inhibition of its ubiquitination.

Our work summarized in Figure 1C showed that in most cells NIR is primarily nucleolar, with only a fraction being nucleoplasmic, while MDM2 is typically in the nucleoplasm. However, occasionally cells were found with strong MDM2 staining of the nucleolus, and it was known that some MDM2-interacting proteins with preference for the nucleolus may translocate MDM2 into that organelle. A reference case is the tumour suppressor protein ARF. Overproduced ARF can bind to MDM2’s central domain and sequester MDM2 in the nucleolus (32,33). It was therefore interesting to ask whether NIR can act in a similar manner. When H1299, U2OS or HeLa cells were transfected to produce HA-tagged MDM2 or Flag-tagged NIR, it turned out that HA-MDM2 was almost exclusively nucleoplasmic, with the nucleoli spared, whereas Flag-NIR was nucleolar (Supplementary Figure S1, panels 1 and 2), as expected and in accordance with the localization of the endogenous proteins (Figure 1C). In contrast, when both proteins were coexpressed, HA-MDM2 like Flag-NIR was almost exclusively present in the nucleolus (Supplementary Figure S1, panels 3, 4). Thus, NIR, like ARF reported before (32,33), can translocate MDM2 into the nucleolus when overproduced, in a manner that is independent of p53. However, it is unclear at present whether this nucleolar translocation is implemented under physiological conditions.

### MDM2 can ubiquitinate NIR and affect its cellular level

**Figure 5.**
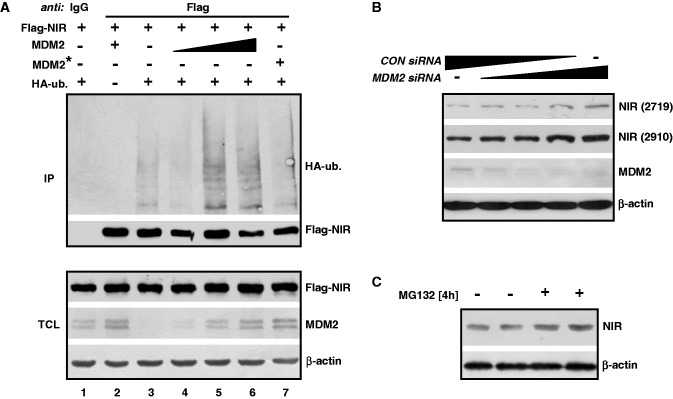
MDM2 ubiquitinates NIR and affects the level of endogenous NIR protein. (**A**) MDM2 with a functional RING domain ubiquitinates Flag-NIR. H1299 cells were transfected to produce Flag-NIR (2 µg), MDM2 (0.1, 0.25 or 0.5 µg), MDM2 RING domain mutant G448S/C449A (MDM2*; 0.5 µg) and HA-ubiquitin (3 µg) for 24 h, after which the cultures were treated with MG132 (10 µM) for another 4 h. Flag-NIR was then immunoprecipitated with anti-Flag antibody or irrelevant IgG from denatured cell extracts and the slower migrating signals indicative of HA-ubiquitinated NIR, as well as the precipitated Flag-NIR and the INPUT protein levels were visualized by western blotting. Anti-Flag monoclonal antibody (1:10 000), monoclonal MDM2 antibody 3G9 (1:2000), anti-HA monoclonal antibody (1:1000) and β-actin antibody (1:10 000) were used for protein detection. TCL, total cell lysate. (**B**) Knockdown of endogenous MDM2 increases the endogenous NIR level. H1299 cells were transfected with control siRNA (*CON siRNA*) and *MDM2 siRNA* (0, 5, 10, 20, 40 ng; each supplemented with *CON siRNA* to 40 ng total siRNA) for 30 h. Total protein extracts were subjected to western blot analysis with polyclonal anti-NIR antibodies 2719 and 2910 (1:200; on the same blot after stripping), and with anti-MDM antibody 3G9 (1:2000) and β-actin antibody (1:10 000). (**C**) Endogenous NIR levels increase after short-term proteasome inhibition. H1299 cells were exposed to MG132 (10 µM) for 4 h and were then analysed by western blotting for the expression of NIR (with antibody 2910) and β-actin as in (B).

Since MDM2 and NIR directly interact it was interesting to ask whether NIR can be a substrate of MDM2. To begin to investigate this, H1299 cells were transfected with plasmids producing Flag-NIR, wild-type MDM2 or MDM2 with a mutated RING domain that was inactive as an ubiquitin ligase but NIR-binding proficient, along with a plasmid expressing HA-tagged ubiquitin. After the treatment of the cells with MG132 to prevent proteasomal degradation, Flag-NIR was immunoprecipitated, again from denatured cell extracts as reported above to only detect HA-ubiquitin that is covalently linked to the precipitated protein, and was analysed by western blotting. The anti-HA antibody produced a signal only when the HA-ubiquitin plasmid was cotransfected (Figure 5A, lane 2). Some HA-ubiquitination of Flag-NIR was already visible in the absence of ectopic MDM2 (lane 3), most likely due to endogenous ubiquitin ligases. HA-ubiquitination of Flag-NIR was increased in the presence of rising levels of ectopic MDM2 (lanes 4–6); however, the HA-ubiquitination could not be increased beyond that level by more MDM2, suggesting that only a portion of NIR was subject to this modification. Coexpression of Flag-NIR with mutant MDM2 that was defective for ubiquitination reduced the HA-signal to background (Figure 5A, compare lanes 3 and 7), suggesting that the ubiquitination of Flag-NIR by MDM2 was dependent of a functional RING domain. Combined, these findings suggested that a portion of ectopic NIR was a substrate of MDM2. Moreover, these findings together with the data shown in Figure 4 suggested that although NIR can inhibit the ubiquitination of MDM2 itself, it does not inhibit the ubiquitin ligase activity of MDM2.

Ubiquitination by MDM2 regulates the levels of numerous cellular proteins, among them p53. We next tested whether the gradual knockdown of endogenous MDM2 by increasing quantities of *MDM2 siRNA* in H1299 cells would affect the levels of the endogenous NIR protein. The results are summarized in Figure 5B. Knockdown of MDM2 correlated with elevated levels of NIR protein, as detected with two different polyclonal NIR antibodies, suggesting that the stability of some endogenous NIR is controlled by MDM2. Consistent with this suggestion, treatment of H1299 cultures with the proteasome inhibitor MG132 for 4 h resulted in elevated NIR levels (Figure 5C).

### NIR supports MDM2’s inhibitory action towards p53 as a transcriptional transactivator

**Figure 6.**
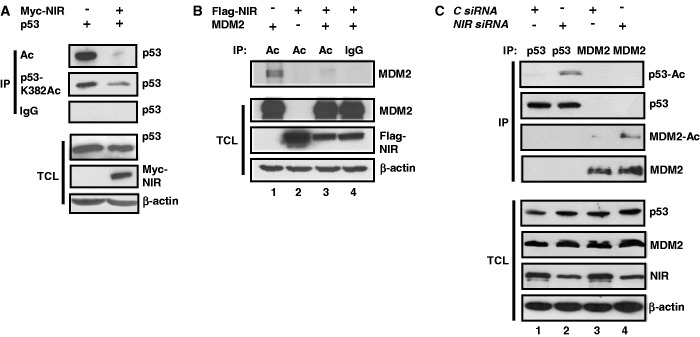
NIR inhibits the acetylation of its protein binding partners. (**A**) NIR inhibits the acetylation of p53. H1299 cells were transfected with p53 (0.4 µg) and Myc-NIR (1 µg) expression plasmid as indicated, and were then treated with MG132 (10 µM) and with HDAC inhibitors trichostatin A (1 nM), sodium butyrate (10 mM) and nicotinamide (5 mM) for another 4 h to block enzymatic deacetylation. Immunoprecipitations were carried out with pan-acetyllysine antibody (Ac), anti-p53 antibody recognizing p53 acetylated at K382 or irrelevant IgG. p53 was detected with anti-p53 antibody DO-1 (1:2000), Myc-NIR with anti-Myc antibody 9E10 (1:2000) and β-actin with its specific monoclonal antibody diluted at 1:10 000. (**B**) NIR inhibits the acetylation of MDM2. HeLa cells were transfected for 24 h with combinations of Flag-NIR (4 µg) and MDM2 (4 µg) plasmids, and were then treated as the H1299 cells in (A). (**C**) Knockdown of NIR correlates with increased acetylation of p53 and MDM2. U2OS cells were transfected with *control siRNA* or *NIR siRNA* (40 nM) as indicated. After 32 h, the cells were treated with ActD (100 nM) and with HDAC inhibitors as specified in (A). Denatured cell extracts were prepared and p53 was precipitated with antibody DO-1, whereas MDM2 was precipitated with a mixture of antibodies 3G9 and 4B11 (5 µg/IP). The western blots were probed with a polyclonal pan-acetyllysine antibody (1:1000), DO-1 (1:2000), 3G9 (1:2000), anti-NIR antibody 2719 (1:1000) and β-actin antibody (1:10 000).

We had reported previously that NIR can bind to p53 and repress gene transactivation (1). Here we presented findings indicating that NIR can also physically and functionally interact with MDM2. However, MDM2, in addition to ubiquitin-marking p53 for degradation, may suppress p53-mediated transcription through the binding to p53 at promoters (20). This binding is posttranslationally regulated. For example, acetylation by the acetyltransferases p300/CBP of central and C-terminal lysines of p53 that are also targets of ubiquitination, is crucial for the sustained separation of p53 from MDM2 (23). Therefore, and since NIR acts as an antagonist of p300/CBP (1), we asked whether NIR can effect the acetylation of p53 and MDM2. To address this, cell cultures were first transfected to produce p53 alone or p53 plus Myc-NIR. At 24 h after transfection, the cultures were treated with HDAC inhibitors for 4 h to inhibit the removal of acetyl groups that had been linked to p53 by cellular acetyltransferases. Total cell extracts were then subjected to immunoprecipitation, either with a pan-acetyl lysine monoclonal antibody, antibody detecting p53 acetylated at K382, or with irrelevant IgG. Western blotting revealed that less pan-acetylated p53, and also less p53-K382Ac, was precipitated in the presence of NIR although the total levels of p53 were similar (Figure 6A). Likewise, when cultures were transfected to express either MDM2 alone or MDM2 together with Flag-NIR, less acetylated MDM2 was precipitated when Flag-NIR was coexpressed (Figure 6B, lanes 1 and 3). These findings suggested that NIR may not only act as an INHAT towards histones (1) but also towards p53 and MDM2.

Next we sought to determine whether siRNA-mediated knockdown of NIR would exert an effect on the acetylation of the endogenous p53 and MDM2 proteins in the presence of the stressor ActD. For this purpose, cells were transfected with control siRNA or NIR siRNA and were again treated with HDAC inhibitors as above, and p53 and MDM2 were then immunoprecipitated from denatured cell extracts and were analysed with an anti-acetyllysine antibody by western blotting. Although the signals that were produced by the acetylated p53 and MDM2 molecules under these conditions were weak, there was consistently more acetylated p53 and MDM2 in the cells in which NIR had been knocked down (Figure 6C, compare lanes 1 and 2 for p53, and lanes 3 and 4 for MDM2), in accord with endogenous NIR being able to inhibit the acetylation of endogenous p53 and MDM2 by p300/CBP. However, since the knockdown of NIR can be detrimental for cells (our unpublished observation; see ‘Discussion’ section) and is therefore likely to cause additional stress, the increased acetylation may simply reflect a reinforced stress response. At present, we cannot distinguish between these possibilities.

**Figure 7.**
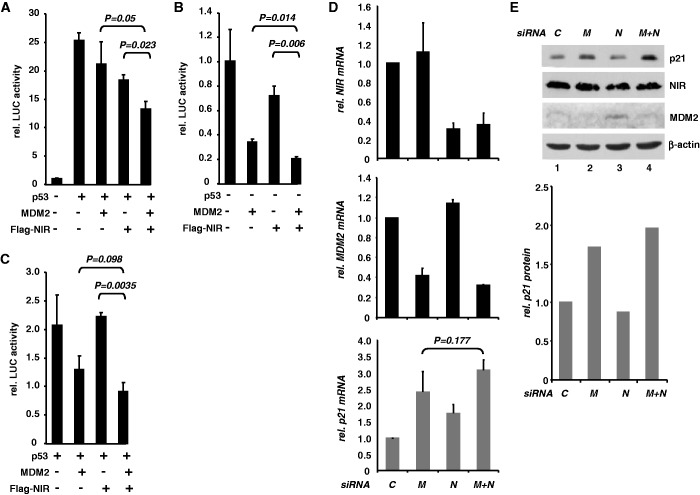
NIR can cooperate with MDM2 to inhibit p53. (**A**) NIR supports the inhibition of p53-mediated reporter gene transactivation by MDM2 in H1299 cells. Cultures were transfected with reporter plasmid PG13-luc (0.3 µg) containing 13 copies of a p53 response element in front of a *luciferase* gene. Cotransfection with p53 plasmid (5 ng) resulted in robust luciferase activity (25-fold activation). MDM2 plasmid and Flag-NIR plasmid were included in the transfections at 75 and 50 ng, respectively. T bars denote standard deviations derived from three experiments; *P*-values were calculated with student’s *t*-test. (**B**) NIR supports the inhibition of p53-mediated reporter gene transactivation by MDM2 in U2OS cells. Cultures were transfected and analysed like the H1299 cultures in (A). Since U2OS cells express wild-type p53, transfection with reporter-vector-only sufficed to produce strong luciferase activity, which was arbitrarily set as 1. T bars denote standard deviations derived from three experiments; *P*-values were calculated with student’s *t*-test. (**C**) Same experiment as in (B), with additional p53 plasmid (5 ng) to increase luciferase activity. MDM2 and Flag-NIR plasmids were used as before. T bars denote standard deviations derived from three experiments; *P*-values were calculated with student’s *t*-test. (**D**) Knockdown of endogenous NIR and MDM2 cooperate to stimulate transcription of the p53-responsive p21 gene. HCT116 cells were transfected with *control* siRNA (*C*), *MDM2* siRNA (*M*), *NIR* siRNA (*N*) or *M*+*N* combined. After 48 h, total RNA was prepared and subjected to RT-qPCR for the quantitation of NIR (upper diagram), *MDM2* (central diagram) and p21 transcripts. The lower diagram (grey bars) shows the p21 transcript levels in response to *NIR* and *MDM2* knockdown. Error bars depict the standard deviations from three experiments. The *P*-value was calculated with Student’s *t*-test. (**E**) Knockdown of NIR and MDM2 cooperate to increase the p21 protein levels. HCT116 cultures were treated as in (D). Total protein extracts were analysed by western blotting with anti-p21 monoclonal antibody (1:1000), NIR antibody 2910 (1:2000), MDM2 antibody 3G9 (1:2000) and β-actin antibody (1:10 000). Densitometry was used to quantitate p21 signal intensity (diagram).

If NIR can inhibit acetylation of p53 and MDM2, and if the absence of acetylation stabilizes the transcriptionally inhibitory p53-MDM2 heteromer (23), NIR might support the suppression of genes by p53-MDM2. To begin to elucidate whether NIR can support MDM2 in the inhibition of p53-mediated transcription, luciferase reporter assays were performed. First, p53-deficient H1299 cells were transiently transfected with a reporter plasmid harbouring p53-response elements driving the *luciferase* gene and with plasmids expressing p53, MDM2 and Flag-NIR. As expected, p53 transactivated the reporter (Figure 7A). MDM2 plasmid was then titrated to the minimal quantity necessary to detectably limit this transactivation. The plasmid producing Flag-NIR was titrated in a similar manner. MDM2 and Flag-NIR individually were able to suppress p53-induced transactivation, as expected (1,20). Notably, MDM2 and Flag-NIR cooperated to suppress transactivation by p53 significantly (Figure 7A). Similar results were obtained when p53-proficient U2OS cells were transfected, regardless of whether ectopic p53 had been included or not (Figure 7B and C). These data are thus consistent with the notion that NIR can support p53-MDM2 in the suppression of p53-mediated transcriptional activation.

Because these experiments were relying on ectopic protein, we next asked whether the effect of cooperativity would be visible on the major endogenous ‘default’ (stress type-independent) p53 target gene *p21* when MDM2 and NIR alone, or both combined, were knocked down by siRNA. Quantitative real-time RT-PCR on p53-proficient HCT116 cells transfected with the different combinations of siRNAs revealed firstly that both MDM2 and NIR knockdowns were efficient and that neither the knockdown of MDM2 had any significant effect on the expression of NIR nor *vice versa* (Figure 7D, top and centre diagrams). Moreover, knockdown of MDM2 resulted in a 2.5-fold increase, and knockdown of NIR in a 1.8-fold increase, in p21 transcript, as expected if MDM2 and NIR act as inhibitors of p53 (Figure 7D, lower diagram). Notably, there was a consistent, albeit non-significant, trend towards elevated p21 expression in cells in which both MDM2 and NIR had been knocked down. Importantly, this trend at the level of p21 transcript was also detectable at the level of p21 protein (Figure 7E): Double-knockdown of MDM2 and NIR led to an increase in p21 protein. Altogether, these findings suggest that NIR and MDM2 can cooperate to inhibit p53.

**Figure 8.**
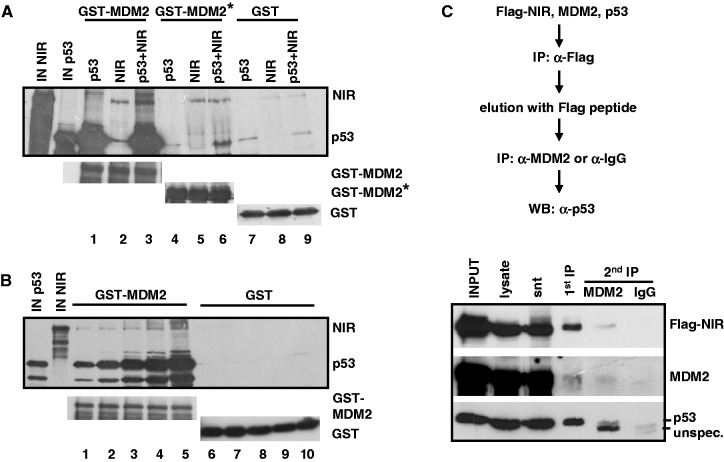
NIR can form a ternary complex with p53:MDM2. (**A**) MDM2 central domain (aa 222–366) that does not bind efficiently to p53 retained p53 in the presence of NIR in pull-down assays. ^35^S-labelled NIR and p53 were incubated with bacterially produced GST, GST-MDM2 or GST-MDM2 222-366 (= MDM2*). IN NIR and IN p53 indicate input controls. Lower panels show the levels of the bacterially expressed GST proteins. (**B**) NIR associates with GST-MDM2 in dependence of prebound p53 in pull-down assays. GST-MDM2 beads were loaded with increasing amounts of labelled p53, and were then incubated with constant amounts of labelled NIR. IN NIR and IN p53 indicate input controls. Lower panels show the levels of the bacterially expressed GST proteins. (**C**) Precipitation of a ternary Flag-NIR:MDM2:p53 complex from transfected cells. The flow diagram outlines the experimental procedure. H1299 cells were transfected to produce Flag-NIR, MDM2 and p53. Flag-NIR and associated proteins were precipitated in a first immunoprecipitation (IP) with anti-Flag antibody (α-Flag) covalently linked to beads. The Flag-NIR complexes were removed from the antibodies with Flag peptide. In a second IP, the complexes were precipitated with a mixture of anti-MDM2 antibodies 3G9 and H221 or incubated with irrelevant antibody (α-IgG). Coprecipitation of p53 was analysed by standard western immunoblotting with the highly sensitive, peroxidase-conjugated anti-p53 antibody DO-1 (1:5000). The other proteins were detected with anti-MDM2 antibody H221 (1:1000) and anti-Flag antibody (1:10 000). snt, supernatant; unspec., unspecific signal.

NIR may help sustain the p53:MDM2 complex by binding to it. The search for a ternary complex was complicated by the fact that all three proteins individually can bind to each of the other two, and that these bindings appear to involve multiple, in part overlapping domains [(1) and Figure 3]. Moreover, complexed NIR, MDM2 and p53 may account for only a small fraction of the total NIR, MDM2 and p53 protein in the cell. All our attempts to detect a ternary NIR:MDM2:p53 complex at promoters *in vivo*, by ChIP-reChIP, have so far been unsuccessful. However, to begin to elucidate whether NIR:MDM2:p53 complexes can exist, we resorted to GST pull-down analyses and sequential immunoprecipitation of cotransfected proteins. First, GST, GST-MDM2 or a GST-MDM2 fragment that consisted of the central acidic and zinc finger domains (residues 222–366) and that bound efficiently to NIR (Figure 3B) but not to p53, were incubated with radiolabelled NIR and p53. While GST alone did not associate with any of the proteins, GST-MDM2 was readily bound by p53 and NIR, as expected. GST-MDM2 (222–366) failed to retain p53 unless NIR was present (Figure 8A). In a further pull-down analysis, a constant amount of GST-MDM2-loaded beads was first saturated for p53 binding by incubation with increasing amounts of labelled p53, and was then incubated with constant amounts of labelled NIR. Consistent with NIR, MDM2 and p53 forming a ternary complex in this *in vitro* assay, the amount of NIR that was retained by the MDM2 beads was dependent on the amount of prebound p53 (Figure 8B). Finally, H1299 cells were transfected to produce Flag-NIR, MDM2 and p53. In a first immunoprecipitation, Flag-NIR and associated proteins were precipitated with anti-Flag antibody covalently linked to beads. The Flag-NIR complexes were then eluted with Flag peptide, and the eluate was subsequently incubated with anti-MDM2 antibodies or irrelevant antibody for a second immunoprecipitation. Western blot analysis ultimately revealed that p53 was coprecipitating with the Flag-NIR:MDM2 complex (Figure 8C). Altogether, we interpret our findings to suggest that NIR can support MDM2-mediated inhibition of p53-driven gene expression by stabilizing MDM2, inhibiting acetylation of p53 and MDM2, and perhaps, by forming a ternary p53:MDM2:NIR complex.

## DISCUSSION

NIR is expressed to high levels in almost all cell lines tested, and NIR is ubiquitously produced throughout murine embryonic development and adulthood (1). Its knockdown in cell lines produces several effects detrimental to the cell, among them the interference with nucleolar rRNA processing; accordingly, all attempts to generate stable NIR-knockdown cell lines by small hairpin RNA (shRNA) expression, and to develop mice with NIR being globally knocked out, have so far failed (our unpublished findings). This together with our observation that NIR is a predominantly nucleolar protein [(8), and Figure 1] suggests that NIR fulfils important functions in that organelle, rather than being merely stored there until becoming mobilized to participate in nucleoplasmic activities. Like many other nucleolar proteins (34), NIR leaves the orderly disintegrating nucleolus during mitosis (8). In the mitotic nucleus it seems to become part of a structure that accompanies the condensed chromatin in metaphase and that is perhaps identical with the so-called perichromosomal region reported before (35). Its function there is unknown but we speculate that it might act as an insulator of the condensed chromatin that keeps histone acetyltransferases in check. However, regardless of cell cycle phase, a fraction of NIR seems to be present in the nucleoplasm all the time (1,8), the compartment where transcription factors such as p53 and p63, as well as their regulators p300/CBP and MDM2, typically reside.

The acetyltransferase p300/CBP is recruited to promoters via p53, and is essential for gene transactivation by p53 in at least three different ways—the block of the inhibitory MDM2-p53 interaction by p53 acetylation (23); the support of p53 DNA binding by p53 acetylation (36,37); and the decompactification of chromatin by histone acetylation (38). It is thus no surprise that NIR, which inhibits p300/CBP-mediated acetylation and can bind to p53, acts as an inhibitor of p53-induced transcriptional activation (1). However, at first glance this inhibitory effect of NIR contrasts with the activities of several other nucleolus-based, p53-regulating proteins that, in response to ribosomal (nucleolar) stress, typically appear to function as activators rather than inhibitors of p53. For example, among the factors that have been reported to leave the stressed nucleolus to resolve the inhibitory p53-MDM2 interaction by modulating MDM2 are the ribosomal proteins L5, L11, L23, L26, S3, S7, S14 and S27/L [reviewed in (27)]. Interestingly, the interaction of most of these proteins with MDM2 is via MDM2’s intrinsically unstructured central acidic domain and is regulated through posttranslational modifications; however, although all these proteins make contact with the same domain, they in fact interact with distinct subdomains, and this specific interaction is thought to be a determinant of their differential effects on MDM2. For instance, whereas ARF and L11 do not inhibit MDM2 ubiquitination, L5 and L23 do (39,40). NIR also associates with the central acidic domain of MDM2, and like L5 and L23 can inhibit MDM2 ubiquitination (Figure 4), though it does not seem to block the enzymatic activity of MDM2 altogether, as suggested by our finding that at least a fraction of cellular NIR can itself be substrate of ubiquitination by MDM2 (Figure 5). Finally, the consequences of the interaction of the ribosomal proteins with MDM2 on the one hand, and of NIR with MDM2 on the other hand, are different. While the ribosomal proteins inactivate MDM2 and stabilize p53 to stimulate p53-dependent transcription (27), NIR inhibits p53 (1) and, as the findings presented here suggest, may support MDM2 to suppress p53-mediated transcription. This suggests that both mechanisms—p53-activation by ribosomal proteins and p53-inhibition by NIR—are implemented in different contexts, and indeed, the release of ribosomal protein L11 and NIR from the nucleolus that was observed in this study in response to the nucleolar stressor ActD (Figure 1) was different.

Low-dose ActD (10 nM) as a stressor sufficed to release L11, as reported before (25), whereas higher doses (≥100 nM) were necessary for the release of NIR. This difference may reflect the distinct effects of these ActD doses on RNA polymerase I and the cell cycle (24). Low doses of ActD are known to primarily interfere with rDNA transcription in the nucleolus because rDNA is GC-rich and ActD binds to guanine residues. This is thought to then trigger, through unknown mechanisms, the release of L11, perhaps together with other proteins that are associated with it in preassembled complexes. High doses of ActD, by contrast, are known to increase the number of cells in G2/M phase of cell cycle (24), and perhaps important in this context, we had reported previously that NIR tends to leave the nucleolus in G2/M phase (8). So if L11 and NIR respond differently to ActD (and perhaps to other forms of nucleolar damage), the underlying mechanisms may be linked to different functional claims on p53 during these distinct responses, reflected by the activation of p53 by L11 as one end point, and by the repression of p53 by NIR as another. It is striking that both effects may involve MDM2.

While the effect of MDM2 on p53 stability has been intensely studied [recently reviewed in (41)], the mechanisms of transcriptional inhibition by the p53-MDM2 complex are less well understood. Important players in the repression of genes by the p53-MDM2 heteromer seem to be the histone acetyltransferases p300/CBP. The acetylation of p53 by p300/CBP is crucial for p53’s function as a transactivator (36,37), and MDM2 can inhibit p53 acetylation by p300/CBP through several mechanisms: (i) the shielding of p53 from p300/CBP interaction (42); (ii) the interference with p300/CBP function in a trimeric p53:MDM2:p300/CBP complex (43); and (iii) the recruitment of deacetylases into the p53-MDM2 complex (44). The findings reported here point to yet another mechanism of inhibition, the recruitment of NIR by p53-MDM2. In this complex, NIR may support the inhibitory p53-MDM2 molecule through at least two mechanisms. First, it may help sustain a compact chromatin structure by blocking histone acetylation by p300/CBP (1). Second, it may stabilize the repressive p53-MDM2 complex through the inhibition of p53 acetylation (23). p53-MDM2 has been shown to reside on the promoters of several p53-regulated genes in non-stressed cells, including the *p21* gene, perhaps as part of a default repressed state of these genes (23). We reported previously that temporary depletion of NIR by siRNA causes a p53-dependent increase in histone acetylation and transcription of the *p21* gene (1). However, although our new data presented here are compatible with the existence of ternary p53:MDM2:NIR complexes (Figure 8), so far our attempts to identify NIR, by ChIP-reChIP, in such complexes on the *p21* gene have failed, perhaps because these complexes are transient due to their restriction to cell cycle phases or other cellular contexts.

In normal non-stressed cells, MDM2 was mostly nucleoplasmic, whereas NIR was mostly nucleolar [(8) and Figure 1C]. On the other hand, overproduction of transfected NIR resulted in the translocation of both proteins to the nucleolus (Supplementary Figure S1). This finding is reminiscent of what has been reported about other MDM2-binding nucleolar shuttle proteins such as ARF (32,45) and might suggest that the interaction/translocation of NIR:MDM2 is typically inhibited in the cells by mechanisms that can be overrun by excess protein. Such hypothetical mechanisms could be subject to regulation during normal cell homeostasis. Thus, in the absence of ectopic protein overproduction, as yet undefined physiological settings may indeed colocalize NIR and MDM2 in the nucleolus. Previous work by Rubbi and Milner has let to the proposal of the nucleolar disruption model of p53 activation (46). The model suggests that the intact unstressed nucleolus actively suppresses the p53 response. Perhaps NIR, rather than being part of the nucleolar stress pathway like many of the ribosomal proteins, is part of an intact nucleolus’ mechanisms to restrain p53. This function of NIR could reach beyond the mere direct inhibition of p53 (1), by supporting the inhibitory p53-MDM2 heteromer through the interaction with both p53 and MDM2.

## SUPPLEMENTARY DATA

Supplementary Data are available at NAR Online.

## FUNDING

The German Research Foundation grants [RO 1201/11-1 to K.R. and SCHU 688/8-1 to R.S.]; HOMFOR (to K.R.). Funding for open access charge: German Research Foundation (DFG).

*Conflict of interest statement*. None declared.

## Supplementary Material

Supplementary Data
