# Supplementary material for: Transcriptional repressor NIR interacts with the p53-inhibiting ubiquitin ligase MDM2
Source: Nucleic Acids Res. 2014 Jan 10;42(6):3565–79. doi: 10.1093/nar/gkt1371 (PMC3973334; doi:10.1093/nar/gkt1371)
Supplement: Supplementary Data [file supp_42_6_3565__index.html]

Transcriptional repressor NIR interacts with the p53-inhibiting ubiquitin ligase MDM2 — Transcriptional repressor NIR interacts with the p53-inhibiting ubiquitin ligase MDM2 — Supplementary Data 

# Transcriptional repressor NIR interacts with the p53-inhibiting ubiquitin ligase MDM2

## Supplementary Data

files

**Files in this Data Supplement:**

- Supplementary Data - doc file
