# Supplementary material for: Transcriptional repressor NIR interacts with the p53-inhibiting ubiquitin ligase MDM2
Source: Nucleic Acids Res. 2014 Jan 10;42(6):3565–79. doi: 10.1093/nar/gkt1371 (PMC3973334; doi:10.1093/nar/gkt1371)
Supplement: Supplementary Data [file supp_gkt1371_nar-03357-x-2013-File010.doc]

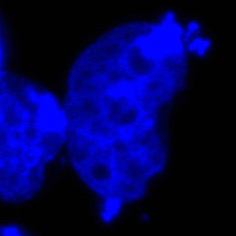

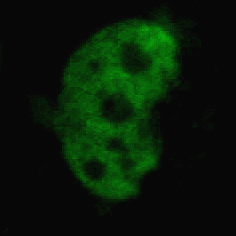

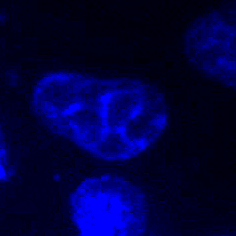

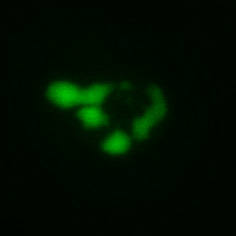

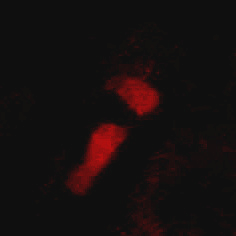

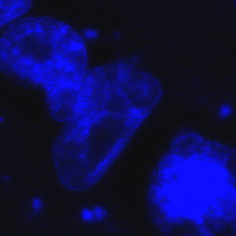


**H1299**

**N M N+M N+M**


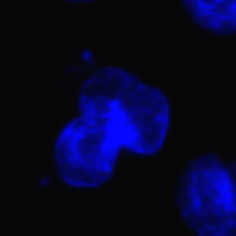

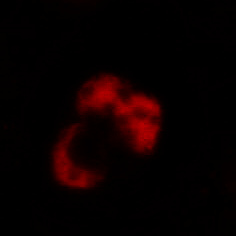


**U2OS**


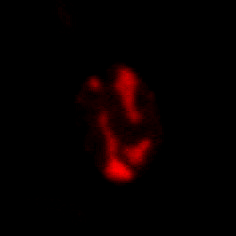

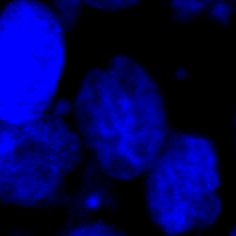

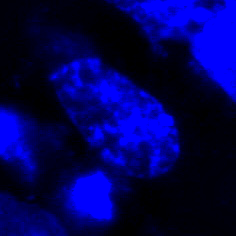

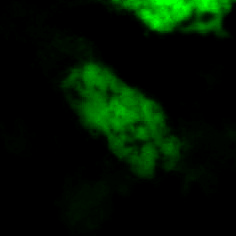

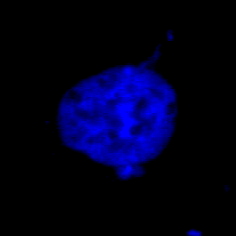

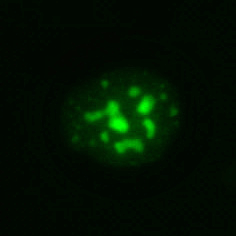

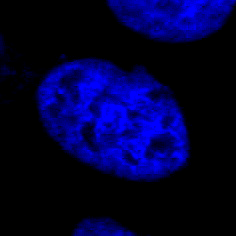

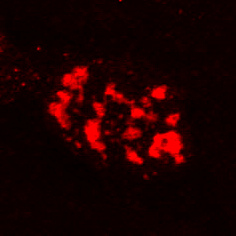

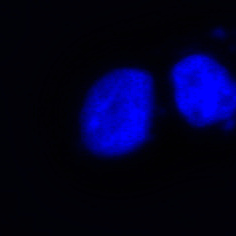

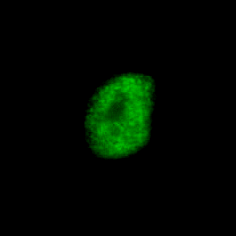

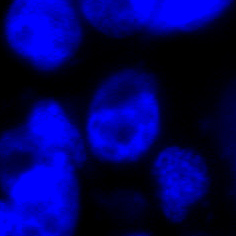

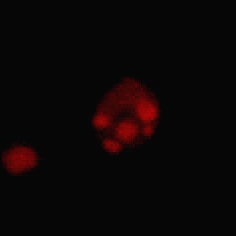

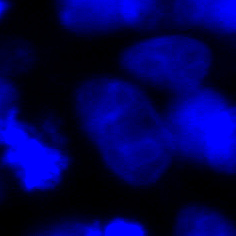

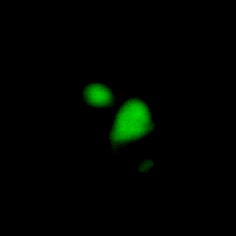

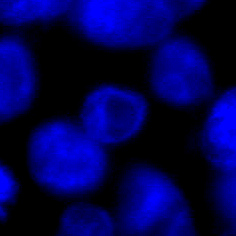

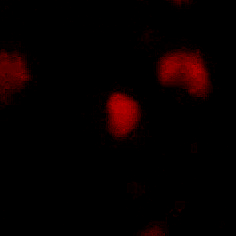


**HeLa**

**1 2 3 4**

**Supplementary Figure 1.** Nucleolar translocation of MDM2 in the presence of NIR. Immunofluorescence analysis of H1299, U2OS and HeLa cells transfected for 24 h with Flag-NIR (N; 1 μg), HA-MDM2 (M; 1 μg) or both (N+M). Cells were treated as described in the legend of Figure 1. HA-MDM2 was detected with a TRITC-conjugated anti-HA antibody, Flag-MDM2 with a Cy3-conjugated anti-Flag antibody. DAPI was employed to stain the nuclei.
